# A Prospective Study of Asymptomatic Intracranial Atherosclerotic Stenosis in Neurologically Normal Volunteers in a Japanese Cohort

**DOI:** 10.3389/fneur.2016.00039

**Published:** 2016-03-22

**Authors:** Ryukichi Matsui, Tomonori Nakagawa, Hiroyuki Takayoshi, Keiichi Onoda, Hiroaki Oguro, Atsushi Nagai, Shuhei Yamaguchi

**Affiliations:** ^1^Department of Neurology, Faculty of Medicine, Shimane University, Izumo, Japan; ^2^Department of Neurology, Masuda Red Cross Hospital, Masuda, Japan; ^3^Department of Laboratory Medicine, Faculty of Medicine, Shimane University, Izumo, Japan

**Keywords:** asymptomatic brain lesions, asymptomatic intracranial atherosclerotic stenosis, risk factors, longitudinal study, magnetic resonance angiography, prognosis

## Abstract

Atherosclerotic stenosis of major intracranial arteries is a leading cause of ischemic stroke in Asia. However, the long-term prognosis of asymptomatic intracranial atherosclerotic stenosis (ICAS) in healthy volunteers has not been fully examined. Here, we conducted a longitudinal study to examine the prognosis of healthy volunteers with asymptomatic ICAS and to determine the risk factors for ICAS, including asymptomatic brain parenchymal lesions. We studied 2,807 healthy Japanese volunteers with no history of stroke (mean age, 62.0 years). They were followed for a mean interval of 64.5 months. The degree of ICAS and the presence of asymptomatic brain lesions were assessed by using magnetic resonance imaging. Asymptomatic ICAS was detected in 166 volunteers (5.9%) at the initial examination. Moderate and mild stenoses were observed in 1.5 and 4.4% of patients, respectively. Significant risk factors for ICAS were older age and a history of hypertension and/or dyslipidemia. During follow-up, ischemic stroke developed in 32 volunteers. Seven strokes occurred in the ICAS group, whose stroke incidence rate was higher than that in the non-ICAS group (0.78 vs. 0.18% per year). According to a Cox regression analysis, asymptomatic ICAS was an independent risk factor for future ischemic stroke after adjustment for age. Furthermore, after asymptomatic brain lesions were taken into account, ICAS was still a significant risk factor for stroke onset. In conclusion, even mild to moderate asymptomatic ICAS was a significant risk factor for future stroke, independent of asymptomatic brain lesions, in a healthy Japanese population. Mild to moderate ICAS might be a therapeutic target for stroke prevention.

## Introduction

Intracranial atherosclerotic stenosis (ICAS) of major arteries represents a common cause of ischemic stroke worldwide (1). In patients with ischemic cerebrovascular disease, the presence of ICAS of >50% has a predictive value for future recurrent ischemic stroke (2). The occurrence of ICAS is associated with advanced age, hypertension, diabetes, and dyslipidemia, and its prevalence increases with a number of risk factors (3). Although the control of risk factors can lead to better outcomes in symptomatic ICAS, the significance of asymptomatic ICAS remains a matter of debate (4). Nahab et al. (5) reported that the prevalence of coexisting ICAS was high in stroke patients, but that the risk of stroke from asymptomatic stenosis was low.

Although many studies have investigated the clinical significance of ICAS in symptomatic patients with stroke (6, 7), reports on the significance of asymptomatic ICAS in healthy volunteers over a long follow-up are limited. Takahashi et al. (8) followed 2,924 Japanese volunteers (mean age, 55 years) without any history of stroke for a mean follow-up duration of 63 months. They reported that the total incidence of cerebrovascular events in persons with ICAS was 1.3% per year. In healthy elderly individuals, on the other hand, silent brain infarctions (SBIs) and subcortical white matter lesions were considered strong risk factors for the onset of subsequent stroke (9, 10). As these asymptomatic ischemic lesions are sometimes associated with ICAS, it is important to consider the influence of asymptomatic ischemic lesions as well as of ICAS on stroke onset. There has been no longitudinal study investigating the long-term prognosis of ICAS in combination with the role of asymptomatic brain lesions. We performed a prospective study to examine whether ICAS was associated with subsequent stroke independent of other risk factors, including asymptomatic brain lesions, in addition to general vascular risk factors in a large number of healthy, elderly Japanese patients.

## Materials and Methods

### Subjects

We originally enrolled a total of 3,161 consecutive Japanese volunteers who voluntarily underwent a medical examination of the brain at the Health Science Center in Shimane between December 2000 and December 2010. The inclusion criteria for this prospective study were as follows: no history of neurological or psychiatric disorders, including stroke, no abnormalities on neurological examination, and the provision of informed consent to participate in this study. For the medical examination, detailed clinical assessments, including medical history, laboratory tests, magnetic resonance imaging (MRI) of the brain, and MR angiography (MRA), were performed. The study design, including information acquisition from other sources, was approved by the institutional ethics committee of Shimane University Hospital.

### Diagnosis of ICAS and Silent Brain Lesions

Magnetic resonance imaging was performed with a 1.5-T scanner (Symphony Ultra Gradient, Siemens) and consisted of transverse T1- and T2-weighted imaging (T1WI and T2WI, respectively) and fluid-attenuated inversion recovery (FLAIR). MRA was also performed using a 3-dimensional time-of-flight gradient echo technique for evaluating intracranial arteries. Extracranial portions of internal carotid artery (ICA) were not covered in our MRA protocol. Vascular stenosis of the main intravascular arteries was assessed using the method by Uehara et al. (11): <25% reduction of an arterial diameter was graded as normal; 25–49% reduction as mild stenosis; 50–74% reduction as moderate stenosis; 75–99% reduction as severe stenosis; and no opening as occlusion. The middle cerebral artery (MCA), the intracranial portion of the ICA, the anterior cerebral artery (ACA), the posterior cerebral artery (PCA), and the basilar artery (BA) were evaluated.

Silent brain infarction, periventricular hyperintensity (PVH), and the presence of deep and subcortical white matter lesions (DSWMLs) were also assessed. SBI was defined as a focally hyperintense lesion >3 mm in diameter on T2WI, corresponding to a hypointense lesion on T1WI. DSWML and PVH were evaluated separately, because PVH is found adjacent to the ventricles, whereas DSWML is found away from them. PVH was graded using a scale of 0–4 (12), and we defined grades 3 and 4 as positive PVH. DSWMLs were graded using a scale of 0–3 according to the Fazekas’ grading scale (13), and we defined grades 2 and 3 as positive DSWML. The radiological assessment was performed by trained neurologists (Tomonori Nakagawa, Hiroyuki Takayoshi, and Hiroaki Oguro) on the day of MRA examination, and the diagnosis was confirmed within several days after MRA examination by neuroradiologists who were blinded to patients’ demographic information.

### Follow-up of Volunteers

Follow-up information about the participants’ health conditions was obtained yearly by mail or telephone interview, and the occurrence and date of medical events were checked. When vascular events were suspected, we obtained information regarding the event by questioning physicians in the hospital that the patient had attended for details of the events, including imaging study results. On the basis of the information obtained from these sources, we determined the stroke type, i.e., cerebral infarction, transient ischemic attack (TIA), intracerebral hemorrhage, or subarachnoid hemorrhage. Because we were interested in the occurrence of ischemic events associated with ICAS, we excluded volunteers from the analysis who developed intracerebral hemorrhage or subarachnoid hemorrhage during the follow-up period (*n* = 10). The final assessment was performed in December 2011.

We could not make any contact with 344 volunteers during the follow-up period. As a result, we analyzed data from a total of 2,807 volunteers (1,497 men and 1,310 women; mean age, 62.0 ± 8.5 years), resulting in an 88.8% follow-up rate. The mean follow-up period was 64.5 ± 30.1 months, ranging from 10 to 132 months. We confirmed no difference in the prevalence of ICAS between volunteers who were successfully followed and those who were lost to follow-up. The selection of the optimal medical therapy for vascular risk factors, including antithrombotic agents, was at the discretion of the patients’ family physicians.

### Background Evaluation

Clinical information obtained included age, sex, history of hypertension (defined by the use of an antihypertensive agent, systolic blood pressure ≥140 mmHg, or diastolic blood pressure ≥90 mmHg), diabetes mellitus (defined as a fasting blood glucose level ≥126 mg/dL, HbA1c ≥ 6.5%, or a history of treatment for diabetes mellitus), and dyslipidemia (defined as a low-density lipoprotein cholesterol level ≥140 mg/dL, triglyceride level ≥150 mg/dL, high-density lipoprotein cholesterol level <40 mg/dL, or a history of treatment with lipid-lowering medication). A smoker was defined as any subject whose smoking index exceeded 200. Regular alcohol consumption was defined as more than 58 mL of alcohol consumed per day.

### Statistical Analysis

Group differences were analyzed using the Student’s *t*-test or the chi-squared test. In order to adjust for age and confounding factors, a logistic regression analysis or an analysis of covariance was performed for the demographic and MRI data. Comparisons of cumulative event-free rates for volunteers with or without ICAS were done using Kaplan–Meier curves with the log-rank test. Cox proportional hazards ratios (HRs) were fitted to ICAS data after adjusting for age and other potentially confounding factors, including drinking, smoking, hypertension, diabetes, dyslipidemia, SBI, DSWML, and PVH. The end point was defined as the occurrence of a cerebrovascular event, including cerebral infarction and TIA. A level of *P* < 0.05 was accepted as statistically significant. Statistical analysis was performed with the SPSS software package (version 22, IBM Corp., Armonk, NY, USA).

## Results

Asymptomatic ICAS was observed in 166 volunteers (5.9%), of whom 42 had moderate and 124 had mild ICAS. In total, stenosis was found in 110 MCA sites, 60 ICA sites, 8 ACA sites, 13 PCA sites, and 14 BA sites. There was neither severe ICAS nor occlusion in this study population. The demographic data and MRI findings in the volunteers with and without ICAS at baseline are shown in Table 1. The volunteers with ICAS were significantly older than those without ICAS. Histories of hypertension, diabetes mellitus, and dyslipidemia were all more prevalent in the volunteers with ICAS compared to such histories in those without ICAS. In addition, all asymptomatic brain lesions, including SBI, PVH, and DSWML, were also highly prevalent among volunteers with ICAS (Table 1). We performed a multiple logistic regression analysis to determine the risk factors for ICAS (Table 2). Age, history of hypertension, and history of dyslipidemia were independent risk factors for ICAS.

**Table 1:** **Demographic and magnetic resonance imaging data in volunteers with and without intracranial atherosclerotic stenosis**.

	ICAS−	ICAS+	*P* value
Number of volunteers	2,641	166	
Age (years)	61.8 ± 8.4	65.6 ± 9.0	<0.001
Sex (M/F, %)	53.1/46.9	56.6/43.4	n.s.
Hypertension (%)	34.4	53.6	<0.001
Diabetes mellitus (%)	9.2	14.5	0.01
Dyslipidemia (%)	46.3	56.6	0.02
Alcohol habit (%)	38.0	40.4	n.s.
Smoking (%)	15.1	12.7	n.s.
SBI (%)	12.7	26.5	0.006
DSWML (grade ≥2) (%)	15.4	31.9	<0.001
PVH (grade ≥3) (%)	3.6	12.7	<0.001

*ICAS, intracranial atherosclerotic stenosis; SBI, silent brain infarction; DSWML, deep subcortical white matter lesion; PVH, periventricular hyperintensity; n.s., non-significant*.

**Table 2:** **Multiple logistic regression analysis of risk factors for intracranial atherosclerotic stenosis**.

	Odds ratio	95% CI	*P* value
Age	1.05	1.03–1.07	<0.001
Hypertension (yes)	1.78	1.28–2.46	<0.001
Diabetes mellitus (yes)	1.41	0.88–2.23	n.s.
Dyslipidemia (yes)	1.53	1.11–2.11	0.009

*95% CI, 95% confidence interval*.

During a mean follow-up period of 64.5 months, 32 (1.1%) volunteers had a cerebrovascular event: ischemic stroke in 26 (0.89%) volunteers and TIA in 6 (0.21%) volunteers. Seven volunteers developed ischemic stroke from the ICAS group at baseline, and the incident rate was 0.78% per year. Three and four volunteers developed stroke in the moderate and mild ICAS groups, respectively. Their stroke incidence rates were 1.33 and 0.6% per year, respectively. In the ICAS group, all stroke lesions were located in the intracranial arteries with stenosis with the exception of one case, in which the lesion was located on the opposite side of the stenotic artery. Twenty-five volunteers did not show ICAS at baseline, and the stroke incidence rate for this group was 0.18% per year. The subject’s characteristics for the stroke and non-stroke groups are shown in Table 3. An older age, a history of hypertension, and a higher prevalence of asymptomatic brain lesions (SBI and DSWML) were associated with ischemic stroke events. The prevalence of ICAS at baseline was significantly higher in volunteers who developed ischemic stroke events than it was in those who did not after adjusting for age.

**Table 3:** **Demographic and magnetic resonance imaging data at baseline in volunteers with and without ischemic stroke events during follow-up**.

	Ischemic stroke (−)	Ischemic stroke (+)	*P* value
Number of volunteers	2,775	32	
Age (years)	61.9 ± 8.5	67.3 ± 7.7	<0.001
Sex (M/F, %)	53.3/46.7	59.4/40.6	n.s.
Hypertension (%)	35.2	59.4	0.008
Diabetes mellitus (%)	9.4	12.5	n.s.
Dyslipidemia (%)	46.9	50.0	n.s.
Alcohol habit (%)	38.0	50.0	n.s.
Smoking (%)	15.0	9.4	n.s.
SBI (%)	13.3	31.3	0.007 0.069^†^
DSWML (grade ≥2) (%)	16.0	46.9	<0.001 0.003^†^
PVH (grade ≥3) (%)	4.0	9.4	n.s.
ICAS (%)	5.7	21.9	0.002 0.005^†^

*SBI, silent brain infarction; DSWML, deep subcortical white matter lesion; PVH, periventricular hyperintensity; ICAS, intracranial atherosclerotic stenosis*.

*^†^*P* value was adjusted for age*.

**Figure 1:**
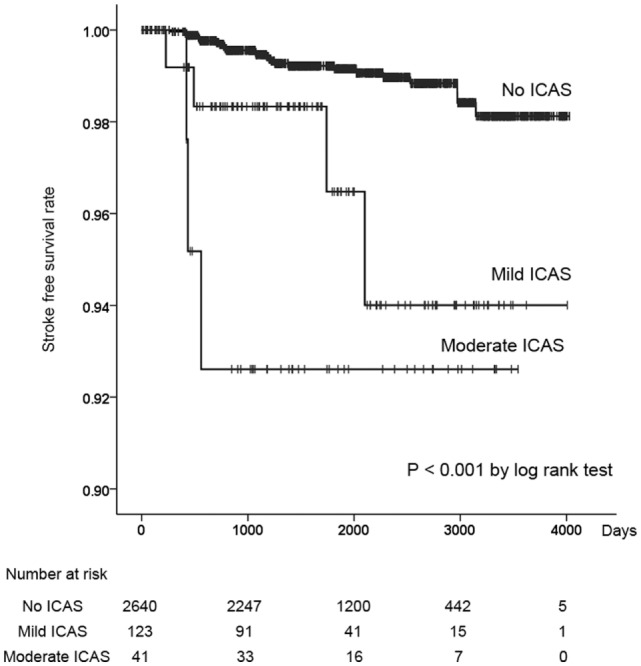
**A Kaplan–Meier survival curve showing cumulative rates free from ischemic stroke events for volunteers with no intracranial atherosclerotic stenosis (ICAS), mild ICAS, and moderate ICAS**.

We performed a Kaplan–Meier analysis with the log-rank test for volunteers with or without ICAS. There was a significantly higher incidence of ischemic stroke events in volunteers with mild or moderate ICAS compared to that in those without ICAS (*P* < 0.001, Figure 1). In the Cox regression analysis, volunteers with mild or moderate ICAS had significantly higher incidences of ischemic stroke events than those without ICAS after adjusting for age [HR, 3.04; 95% confidence interval (CI), 1.17–7.43; *P* = 0.042 for mild ICAS; HR, 6.10; 95% CI, 1.83–20.4, *P* = 0.003 for moderate ICAS]. There was no significant difference in stroke incidence between volunteers with mild and those with moderate ICAS. Furthermore, we assessed the influence of asymptomatic brain lesions (i.e., DSWML and SBI) on ICAS contribution to stroke events, because the asymptomatic MRI lesions were frequently associated with ischemic stroke events (Table 3). Data regarding asymptomatic MRI lesions were incorporated into multiple regression models in a stepwise fashion after adjusting for age. The following variables were used for each model: the presence of ICAS for Model 1, the presence of ICAS and DSWML for Model 2, and the presence of ICAS, DSWML, and SBI for Model 3. As seen in Table 4, ICAS was still a significant independent risk factor for ischemic stroke events after adjusting for DSWML and SBI (Models 2 and 3 in Table 4).

**Table 4:** **Logistic regression analysis results for the occurrence of ischemic stroke events**.

Variables	HR (95% CI)		
	Model 1	Model 2	Model 3
ICAS (0 = no, 1 = yes)	3.54^§^ (1.47–8.51)	3.06^†^ (1.26–7.46)	2.94^†^ (1.20–7.18)
DSWML (grade ≥ 2) (0 = no, 1 = yes)		2.97^‡^ (1.36–6.48)	2.77^†^ (1.25–6.13)
SBI (0 = no, 1 = yes)			1.60 (0.71–3.60)

*All analyses were adjusted for age*.

*HR, hazards ratio; 95% CI, 95% confidence interval; ICAS, intracranial atherosclerotic stenosis; DSWML, deep subcortical white matter lesion; SBI, silent brain infarction*.

*^†^*P* < 0.05*.

*^‡^*P* < 0.01*.

*^§^*P* < 0.005*.

## Discussion

Results from the current longitudinal cohort study demonstrated that even mild to moderate asymptomatic ICAS was an independent risk factor for future ischemic stroke in a healthy population. Many studies have confirmed that there is a high risk of vascular events in patients with symptomatic ICAS who had already experienced stroke. In a Chinese multicenter study, a higher frequency of recurrent stroke was associated with increasing severity of ICAS (14), indicating the importance of the degree of intracranial vascular stenosis. Wong and Li (15) reported an annual recurrent stroke rate of 17.1% for patients with symptomatic ICAS. On the other hand, the prognosis of asymptomatic ICAS has not been fully examined, especially in healthy volunteers. Kremer et al. (16) studied 50 Caucasian patients with asymptomatic atherosclerotic MCA stenosis (MCAS) and concluded that asymptomatic MCAS appeared to have a benign long-term prognosis with a low risk of ipsilateral stroke. Another study compared the prognosis of symptomatic and asymptomatic MCAS and reached a similar conclusion (12.5% for symptomatic ICAS and 2.8% for asymptomatic ICAS) (17). Furthermore, a recent hospital-based study reported that the recurrence rate of cerebrovascular events was 12.3% per year for symptomatic ICAS and 0.88% per year for asymptomatic ICAS (18). This incidence rate of stroke from asymptomatic ICAS was similar to that found in our study (0.78% per year). Even though stroke incidence from asymptomatic ICAS is relatively low, our study showed that the stroke incidence in the ICAS group was significantly higher compared to that in the non-ICAS group (0.18% per year). Thus, incidental identification of asymptomatic ICAS in healthy volunteers should not be ignored, even when the stenosis is mild.

Elderly people with SBI and DSWML have been reported to be at a highly increased risk of stroke, which could not be explained by other major stroke risk factors (9, 10). Park et al. (19) reported that symptomatic ICAS was independently associated with progressively greater DSWML burden, and the association of ICAS with DSWML severity appeared to be stronger than that of the relationship of DSWML severity with extracranial atherosclerotic stenosis. That study indicated that symptomatic ICAS was associated with DSWML, but it is not clear how much asymptomatic ICAS affects the emergence of asymptomatic brain lesions, such as SBI and DSWML. We conducted a logistic regression analysis that incorporated asymptomatic brain lesions into the regression models, and the results showed that asymptomatic ICAS was still a significant predictor for ischemic stroke events. The current study demonstrated that asymptomatic ICAS and DSWML independently contributed to future stroke occurrence. To the best of our knowledge, this is the first study to demonstrate asymptomatic ICAS as an independent risk factor for future stroke, even in healthy volunteers, after adjusting for asymptomatic brain lesions, which are strong risk factors for ischemic stroke.

The control of risk factors for ICAS is important for preventing stroke associated with ICAS lesions, because most risk factors are treatable. The current study demonstrated that age, hypertension, and dyslipidemia were independent risk factors for asymptomatic ICAS. Many studies have investigated the risk factors for asymptomatic and symptomatic ICAS. Histories of lipid disorder and metabolic syndrome were common in patients with severe intracranial stenosis in the warfarin vs. aspirin for symptomatic intracranial disease (WASID) trial (7). Age and diabetes were independently associated with moderate to severe ICAS in the Barcelona-AsIA study (20). A long-term longitudinal study with medical intervention would be necessary in order to determine the optimal strategy for preventing stroke onset in patients with asymptomatic ICAS.

The prevalence of asymptomatic ICAS was 5.9% in this Japanese cohort. Many studies have confirmed that extracranial carotid atherosclerotic stenosis is the most common vascular lesion found in Caucasian stroke patients, while intracranial atherosclerotic disease is found commonly among stroke patients of Asian, African-American, and Hispanic ancestry (21, 22). In previous Asian studies using transcranial Doppler imaging, the prevalence of asymptomatic intracranial atherosclerosis ranged from 6 to 24.5% depending on the age and degree of vascular risk in recruited volunteers (3, 11, 23, 24). The discrepancy in the prevalence rate among studies might be due to differences in the number of risk factors and kind of assessment tools. The low prevalence of ICAS in our study may be attributed to the lower number of risk factors compared to that in studies for high-risk patients.

An optimal medication has not been found for ICAS patients. Antithrombotic therapy for intracranial arterial stenosis was evaluated in the WASID trial. The authors reported that the risk of stroke in the territory of the stenotic artery was the highest in patients with severe stenosis (HR, 2.03) (25). In case of asymptomatic ICAS, medical intervention has not been considered because the stroke risk is relatively low. However, because the long-term prognosis in asymptomatic ICAS patients is worse compared to that in non-ICAS volunteers, the stenotic lesions may be a treatment target for such medications as cilostazol, which was reported to improve ICAS lesions (26).

The limitation of this study is that it was not possible to regulate the treatment for vascular lesions and risk factors because our brain examination system was only for medical examination, and the optimal medical therapy, including antithrombotic agents, was at the discretion of the patients’ family physicians. The information of adherence to medical treatment and control status of risk factors was not available. The possibility exists that stroke may have occurred in volunteers under inappropriate medical conditions. However, this is unlikely, because volunteers underwent the brain examination voluntarily and at their own expense. Second, the assessment of vascular stenosis was performed with just one modality (i.e., MRA). Most cohort studies used a Doppler device for detecting MCAS, which is inevitably limited for surveillance because the detection rate of stenosis is dependent on the operator’s skill, and it is difficult to study vessels other than the MCA with Doppler imaging. Thus, direct comparisons of our study with previous cohort studies are difficult. Furthermore, it was not clear whether the development of stroke was associated with progression of vascular stenosis, because the follow-up MRA data were not available. Longitudinal investigation of MRA data would be highly valuable to confirm the significance of ICAS.

In conclusion, our longitudinal prospective study using MRI and MRA demonstrated that even a mild to moderate degree of asymptomatic ICAS is a significant independent risk factor for future ischemic stroke independent of asymptomatic brain lesions in a healthy population. Future studies are needed to improve prognosis in volunteers with asymptomatic ICAS.

## Author Contributions

The contributions of authors are as follows: SY designed the study; RM, TN, HT, HO, and AN collected the data; RM, KO, and SY analyzed the data; RM and SY wrote the paper. All authors read and agreed to the final version of the paper.

## Conflict of Interest Statement

The authors declare that the research was conducted in the absence of any commercial or financial relationships that could be construed as a potential conflict of interest.
